# Gendered knowledge and adaptive practices: Differentiation and change in Mwanga District, Tanzania

**DOI:** 10.1007/s13280-016-0828-z

**Published:** 2016-11-22

**Authors:** Thomas A. Smucker, Elizabeth Edna Wangui

**Affiliations:** Department of Geography, Ohio University, Clippinger Labs 122, Athens, OH 45701 USA

**Keywords:** Agriculture, Climate-change adaptation, Community-based adaptation, Local knowledge, Local institutions, Pastoralism

## Abstract

We examine the wider social knowledge domain that complements technical and environmental knowledge in enabling adaptive practices through two case studies in Tanzania. We are concerned with knowledge production that is shaped by gendered exclusion from the main thrusts of planned adaptation, in the practice of irrigation in a dryland village and the adoption of fast-maturing seed varieties in a highland village. The findings draw on data from a household survey, community workshops, and key informant interviews. The largest challenge to effective adaptation is a lack of access to the social networks and institutions that allocate resources needed for adaptation. Results demonstrate the social differentiation of local knowledge, and how it is entwined with adaptive practices that emerge in relation to gendered mechanisms of access. We conclude that community-based adaptation can learn from engaging the broader social knowledge base in evaluating priorities for coping with greater climate variability.

**Electronic supplementary material:**

The online version of this article (doi:10.1007/s13280-016-0828-z) contains supplementary material, which is available to authorized users.

## Introduction

There is growing recognition of the contributions of local knowledge to both planned and spontaneous adaptation to climate change in developing countries. Local knowledge of agro-ecological variability enables adaptive management and may be the basis for identifying sustainable pathways for future adaptation in agricultural and pastoralist systems (Vignola et al. 2009; Nakashima et al. 2012). Peoples’ means of coping with past extreme climatic events may offer insights into local knowledge-based practices that can be “scaled up” or “scaled out” through planned adaptation (Forsyth 2013; Reid and Schipper 2014). Local knowledge may even refine scientific understanding of the physical characteristics of climate change by providing information at time and spatial scales that are more appropriate to enhance adaptive management as compared to information provided by climate science (Ziervogel et al. 2010; Rao et al. 2011; Rakshit et al. 2014). The IPCC’s “Special Report on Managing the Risks of Extreme Events and Disasters to Advance Climate Change Adaptation” found robust evidence and high levels of agreement in the scholarly literature that the integration of local knowledge with technical and scientific expertise has contributed to risk reduction and effective adaptation to climate change (IPCC 2014).

Despite the optimistic tone prevailing in contemporary research on adaptation to climate change, there remains considerable diversity in conceptualizations of local knowledge and, consequently, uncertainty about the purposes and purported benefits of knowledge integration. In this paper, we ask how applying a gendered lens to the relationship between local knowledge and adaptive practices alters the conventional and largely positive appraisal of this relationship. We present the results of field research on local knowledge contributions to adaptive practices that demonstrate the grounding of local knowledge in local cultural institutions and histories of managing local environmental landscapes. However, the research also demonstrates the dynamic and socially differentiated nature of local knowledge, particularly along gendered lines. In addition to incorporating external knowledge, dynamism is also driven by local experimentation and learning that has enabled adaptive responses to both climatic and social stressors. Specifically, knowledge–practice complexes reflect local responses to widely described shifts in climate variability as well as the limitations imposed by gendered mechanisms of access to land, technology, authority, and knowledge that produce differential vulnerability to climate change.

The term local knowledge is often used interchangeably with indigenous and traditional knowledge and described as distinct and isolated from scientific forms of knowledge. Conventional definitions emphasize the holistic or integrative nature of local knowledge and its accumulation through direct monitoring, evaluation, and response to change in specific environmental contexts (Nakashima et al. 2012). The historical continuity of such systems is sustained through cultural institutions that make possible intergenerational knowledge transmission, particularly through oral traditions (Berkes et al. 2000). A variant of local knowledge, traditional ecological knowledge (TEK) is a longstanding focus of scholars working within resilience and sustainability science frameworks (Singh et al. 2010; McCarter and Gavin 2014). A recurrent theme of this work is the erosion of such knowledge systems as a result of modernization, thereby threatening the resilience of local management systems and, cumulatively, reducing the diversity of knowledge systems that societies will rely on to achieve sustainability or successful adaptation. As with similar research on indigenous knowledge systems, TEK research “evokes embattled ways of living-in-the-world that real economic, social, and political pressures are nudging and frog-marching toward further marginalization and oblivion” (Agrawal 2009, p. 158). The narrative of two unitary and fundamentally distinct knowledge systems—one ever expanding in influence and reach while the other rapidly erodes—has created a sense of urgency with regard to “preserving” local knowledge systems, as further loss erodes the knowledge base for environmental management and climate-change adaptation. Importantly, such framings have largely reinforced an image of local knowledge systems as fundamentally static and internally undifferentiated (Goldman 2007; Ramisch 2014).

In contrast to the narrative of erosion and loss, alternative framings of local knowledge have recast these systems as dynamic, differentiated and by no means merely traditional or technical in nature (Wisner 2010; Naess 2013). Rather than static unitary systems in decline, local knowledge systems are more accurately portrayed as hybrid systems that result from the synthesis of multiple external influences, even as they retain strong cultural- and place-based foundations (Ellen et al. 2000). However, hybrid knowledge is not produced uniformly within communities, but rather it reflects differential exposure to external knowledge sources and different processes of evaluation and synthesis within the context of existing livelihood strategies and struggles (Nygren 1999). Dynamism in local knowledge system may be further driven by local innovation when local change in environmental or social conditions renders existing knowledge and practice less effective (Adger et al. 2013).

This more expansive, dynamic, and heterogeneous framing of local knowledge holds implications for how we understand gendered differences in vulnerability to climate change. It is increasingly recognized that gendered forms of social inequality intersect with other forms of social differentiation, such as class, to shape vulnerability and adaptive capacity to climate change (Sultana 2014). Differential vulnerability to climate change has often been examined through the lens of inequality in rights-based resource access established through formal tenure systems. For example, in many least developed countries such as Tanzania, this primary focus on rights-based access is understandable given longstanding gender inequality in land rights and security of tenure that may constrict livelihood diversification and thereby limit adaptation options (Mbilinyi 2003; Ikdahl 2008; Peterman 2011). Although Tanzania’s new land laws in 1999 sought to explicitly increase women’s land access and security of tenure, women of lower social standing continue to face considerable obstacles in pursuing and maintaining land claims through village councils (Pedersen 2015). In light of this, we argue for a focus on unequal access that encompasses the “wider range of social relationships that constrain or enable people to benefit from resources without focusing on property relations alone,” including structural determinants of access such as knowledge, technologies, and authority (Ribot and Peluso 2003, p. 154). Alongside formal resource rights, gendered control of knowledge and information, and the ability to shape local environmental or development discourses are important to the production of gendered vulnerability (Tuana 2013).

We highlight local knowledge dimensions of climate-change adaptation that have received relatively little attention. First we are concerned with the wider social knowledge domain that complements technical and environmental knowledge in enabling adaptive practices. This framing draws from the notion of Rocheleau et al. (1996, p. 8) of a gendered science of survival through which women “develop and maintain their integrative abilities to deal with complex systems of household, community, and landscape.” Second, we assert that local knowledge systems are also shaped by unequal access to knowledge about rights, resource tenure, and external technologies and practices that emanate from formal institutions (Osbahr et al. 2010; Pettengell 2010). The question of access to multiple kinds of information further underlines the extent to which local knowledge systems are enmeshed in broader webs of access to technologies and authority (Ribot and Peluso 2003).

We draw on evidence from two case studies to rethink the role of local knowledge in adaptation. Both cases draw our attention to knowledge production at the margins that is shaped by exclusion from the main thrusts of rural development and planned adaptation to climate change, in these cases related to the practice of irrigation in a dryland village and the adoption of fast-maturing seed varieties in a highland village. We examine the knowledge-practice dynamics associated with primary adaptive practices undertaken in two communities.

## Study context

**Fig. 1:**
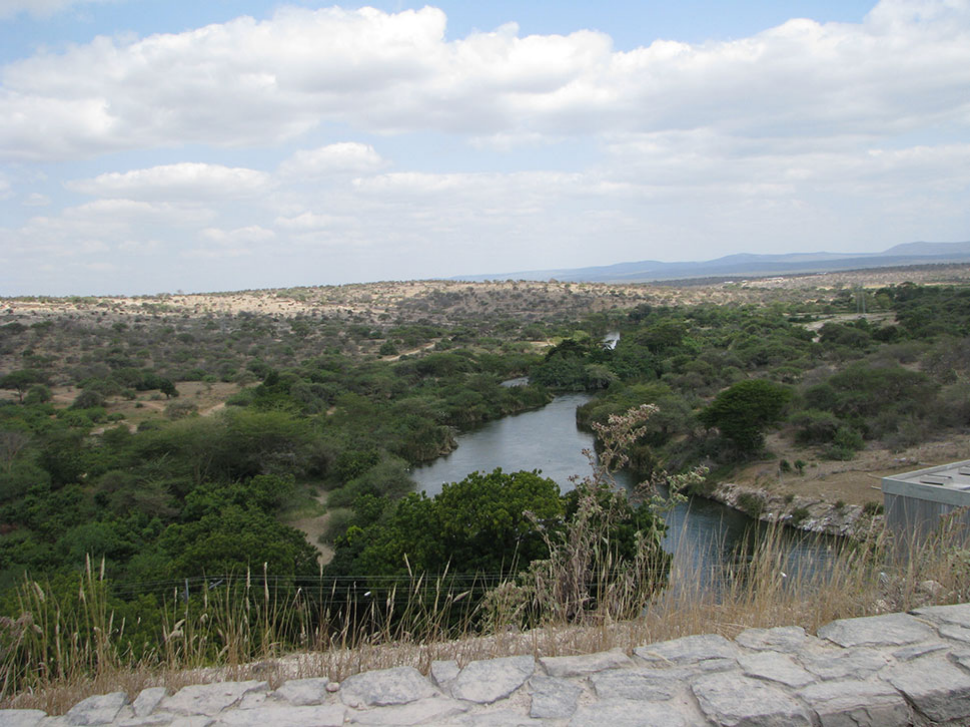
The typical landscape of Kirya Village: A semiarid lowland area in Mwanga District. The photo shows River Pangani (Ruvu) in the foreground (photo by E. E. Wangui)

**Fig. 2:**
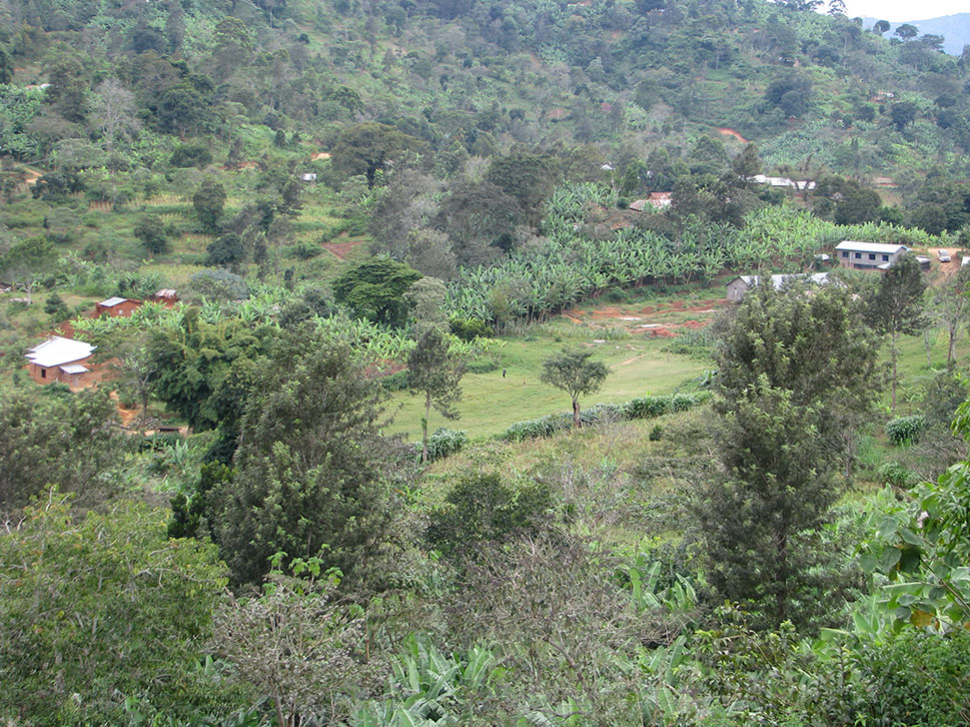
The typical landscape of Mangio Village in the North Pare Mountains (photo by E. E. Wangui)

Mwanga District lies in the Kilimanjaro Region of northern Tanzania. According to The Mwanga District Socioeconomic Profile, the total population of the district was estimated at 142 990 in 2012, spread across 70 villages (URT 2011). The results presented here are from two villages that fall in different ecological and livelihood zones within the district. Kirya Village is in the semiarid lowlands where the main livelihood activity has historically been pastoralism. Mangio Village lies in the humid and sub-humid highlands where farmers rely on a mix of intensive rain-fed and irrigated agriculture made possible by an extensive hill furrow irrigation system. Figures 1 and 2 show the typical landscapes in each of the two villages. A focus on these two diverse landscapes allows us to examine the local knowledge dimensions of adaptive practices in two distinct ecological and livelihood contexts.

**Fig. 3:**
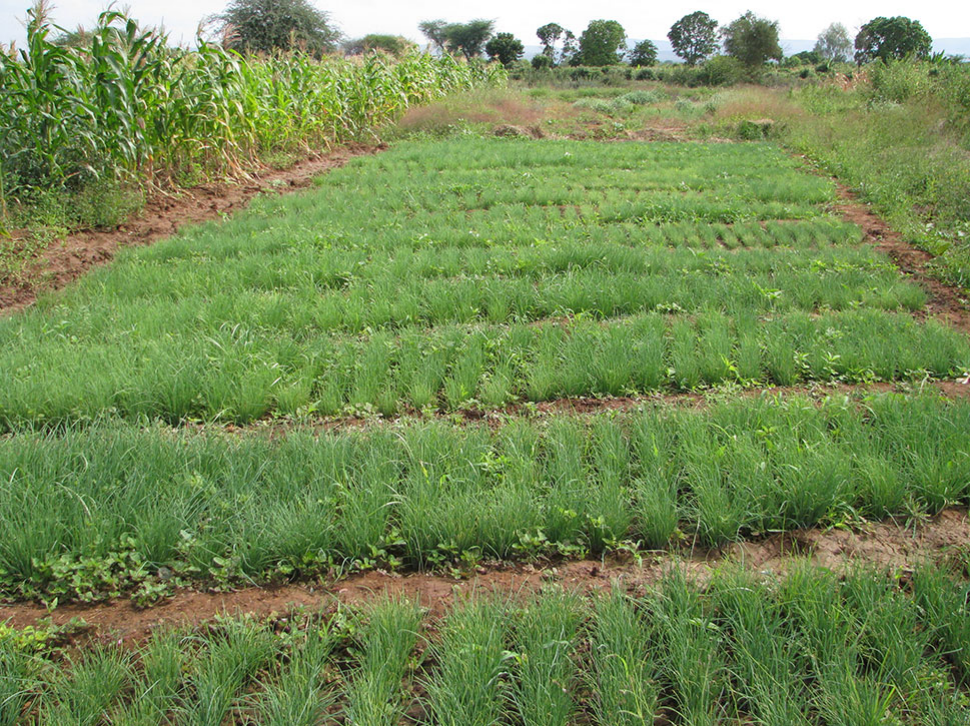
Irrigated onion plot in Kirya village (Photo by E. E. Wangui)

Data from Kirya village administrative offices show that the village has a total of 323 households, 135 of which rely heavily on livestock. According to long term residents of Kirya, Maasai pastoralists were the first to settle permanently in Kirya in the 1950s. They moved to Kirya for grazing purposes. They were followed by Warusha and Pare ethnic groups, who started to divert water from The Pangani River for irrigation as far back as the late 1950s. Migrants from other parts of Tanzania (including Mangio) have continued to move to Kirya to practice irrigation, making the village very ethnically diverse. Today, Kirya Village is home to over thirty different ethnic communities. Data from the village administrative offices indicate that all of the 410 ha of farmed land is under irrigation. Figure 3 shows what a typical irrigated plot in Kirya village looks like. As has been found in other parts of East Africa (Wangui 2008; Homewood et al. 2009), Maasai pastoralists in Kirya have taken up irrigated farming as a form of livelihood diversification. The results presented here are from the Emangulai B sub-village of Kirya, which is occupied only by Maasai pastoralists.

Mangio Village is located in the North Pare Highlands, which form part of the Eastern Arc Mountains that are a biodiversity hotspot (Burgess et al. 2007). The village is occupied almost exclusively by Wapare people who are historically farmers. The 315 households in the village practice rain-fed farming as the main livelihood activity, complemented with irrigation that is practiced in the low-lying areas within the village. Fast growing seed varieties have been available in Mangio since 2009 through donor funded government initiatives. The seeds are predominantly maize, but beans and sunflower seeds are also planted to a smaller extent. Typically, fast growing maize varieties take between 65 and 75 days to reach maturity. Although they are more costly, they have an advantage over other maize varieties that take between 90 and 120 days. Regardless of the type of seeds farmers plant, they do so earlier in the season than they have in the past in response to increasing rainfall variability associated with climate change.

Gender plays an important role in how men and women access assets necessary for climate-change adaptation in both villages (Muthoni and Wangui 2013). Through customary law, men hold disproportionate power in resource access and decision-making. Despite recent land reforms that encourage joint registration of new allocations of village land, most women access land and other productive assets through their male relations. Although the legal framework supports both male and female land ownership, women hold only 20% of the registered land in Tanzania (USAID 2011). Patriarchy is pronounced in both villages and patriarchal structures are partially responsible for the inequality in access to assets. Patriarchy among Maasai pastoralists and farming communities of the North Pare highlands is well documented and will not be explored here (e.g., Hodgson 1999; Sheridan 2002; Gneezy et al. 2009; Grabe et al. 2015).

## Materials and methods

The results presented in this paper are part of a larger five-year project that began in 2010 and examined how local knowledge and local institutions mediate adaptive capacity to climate change. This broader research was conducted along a total of four altitudinal gradients of Mt. Kilimanjaro and North Pare Mountains that extend into neighboring savanna drylands. The research drew broadly from the emerging methodologies of Community Based Adaptation (CBA) (Ensor and Berger 2009; Reid et al. 2009). The research team’s commitment to community engagement led to an interactive and deliberative process of assessment of different kinds of data with participants in community workshops. To meet our research goals for this paper, field research examined the primary adaptive practices in each of the two villages, as well as how these varied within each community. We also looked at how different gendered subjects navigated local institutions that facilitate adaptation to climate change. We therefore collected data at three different levels as explained below.

First, we carried out a community workshop to assess key adaptive practices in each village. We worked with the village leadership to extend a general invitation to community members to participate in the workshop. We followed up this general invitation with targeted invitations to ensure that gender and socioeconomic status were represented at each workshop. In Emangulai B, 12 men and 14 women attended the workshop, while in Mangio 15 men and 4 women were present. We started each workshop with an exercise where community members listed all the adaptive practices undertaken in the village. In a second exercise, participants *ranked* the adaptive practices based on their perceived effectiveness in reducing climate risk. A rank of 1 was given to the practice they perceived to be the most effective. The final exercise at the workshop was an assessment of each adaptive practice based on perceived accessibility within the community. Accessibility was assessed on a five point Likert scale where 1 meant only a few people practiced the activity.

Second, we collected household-level data in order to understand how adoption of the adaptive practices discussed at the community workshops varied within the community. In preparation for the household-level data collection, we carried out a wealth ranking exercise together with key informants who were familiar with everyone living in each village. Together, we identified three wealth groups and sampled 9–12 households in each group. In total, 29 households were interviewed in Emangulai B while 33 were interviewed in Mangio. In each household, we interviewed the key decision maker(s) involved in the adaptive practice. In Emangulai B, 18 men and 11 women were interviewed, one person in each household. A total of 27 men and 25 women were interviewed in the 33 households in Mangio. Each household rated each of the adaptive practices discussed at the community level on a five-point Likert scale based on the perceived effectiveness in reducing the household’s climate risk. One meant that the perceived effectiveness was very low, while 5 meant that it was very high. If a household did not practice the adaptation in question, a score of zero was given. Respondents were then asked to talk about their experience with each adaptive practice. For example, *why don’t you use fast growing seeds? what prevents you from irrigating? Why did you give irrigation/fast growing seeds the score that you did?*

Third, to further deepen our understanding of social differentiation within communities, we conducted key informant interviews to examine how men and women mobilize local knowledge to navigate the institutional context within which climate-change adaptation takes place. In each village, ten key informants (five men, five women) were identified from the household interviews based on how successful they were in utilizing the adaptive practices. We therefore interviewed people who had scored the adaptive practice in question high and those who had scored the practice low. Additional key informants came from the village administration involved in land allocation (in Kirya) and seed distribution (in Mangio). The results presented in this paper are focused on the most effective agriculture-based adaptive practice in each village: Irrigation in Kirya Village (overall rank of 1), and use of fast-maturing seed varieties in Mangio Village (ranked second after education). Both practices are also unique since they have been heavily promoted for climate-change adaptation by the Tanzania government and international donors (United Republic of Tanzania 2012).

All the qualitative data collected at the three levels were transcribed and entered into NVivo qualitative data analysis software. Initially, open coding of whole sentences and paragraphs was completed and specific categories were developed (Strauss and Corbin 2014). The goal of open coding was to identify how local knowledge and local institutions influence climate-change adaptation. Open coding was followed by axial coding where we compared categories and identified conceptual themes within and between interviews. At this stage, analysis focused on questions relating to who, when, where, how, why, and with what consequence. Gender disaggregated averages were calculated from the Likert scale data collected at the household level. Data analysis and data collection were done iteratively during the household and key informant interviews so that important ideas and patterns emerging in each interview could be investigated in subsequent interviews.

## Results and discussion

### Gendered access to land: Irrigation as adaptive practice in Kirya

A full list of adaptive practices discussed in Emangulai B and their respective ranks, accessibility scores and effectiveness at the household can be found in Table S1. Although irrigation was identified in community workshops as the most effective adaptation in Emangulai B (rank of 1), only about half of the families in the community practiced it (score of 3). One constraint to irrigation identified in community workshops and key informant interviews is poorly developed irrigation infrastructure. This was confirmed by our field observation that only areas adjacent to River Pangani have easy access to water for irrigation. The Mwanga District socioeconomic profile indicates that Kirya Village has a potential to irrigate 890 ha (URT 2011). Data obtained from the village office indicate that only an area of 410 ha is actually under irrigation. Given this background, it was not surprising that irrigation was scored quite low by both men (average score of 1.3) and women (average score of 1.6) in terms of its effectiveness in reducing climate risk in their respective household (Table S1). This is particularly telling since irrigation as an adaptation to climate change has been given a high priority by Tanzania’s national government as is evident in national documents such as the National Adaptation Program of Action (NAPA) and *Kilimo Kwanza* (Agriculture First) (Smucker et al 2015). More recently, Tanzania passed the 2013 National Irrigation Act in order to support smallholder farmers such as those in Kirya (URT 2013). Our results indicate that such national-level efforts have done little to make irrigation more accessible in Kirya.

Results from the individual interviews reveal that one of the largest challenges to irrigation is a lack of access to the social networks and institutions that allocate land. In 1999, Tanzania enacted the Village Land Act (VLA) which lays out the legal process through which rural lands are managed (URT 1999; Wily 2003; Dancer 2015). Based on the Act, land tenure and land disputes are managed by the village government, which consists of a village chair and village council. Village land rights are registered through the Customary Rights of Occupancy (CRO). Thus, when the village council allocates land, it issues a CRO. The Act requires the village council to take into account the land rights of women, and to consider the needs of landless people. The VLA was part of a broader process of devolution and decentralization of government decision-making whose goal was to “empower citizens to manage their own affairs” (Wily 2003, p. 2). In practice, the process laid out in the VLA and implemented in Kirya is complex. Many do not understand the administrative process of establishing a right of occupancy and are therefore unable to successfully navigate it.

We found that access to knowledge of the land-allocation process is closely tied to an individual’s social networks and spatial mobility. Both networks and mobility contribute to make land acquisition a highly gendered process. During key informant interviews, respondents explained that men are more likely to travel to the village offices where they were likely to interact with members of the village council, most of whom are men. It is through such formal and informal interactions that many men have gained knowledge about the land-allocation process. As in other rural pastoralist communities, women are primarily involved in reproductive responsibilities and livestock duties that typically do not provide opportunities for travel to village offices or interact with the members of the village council (Wangui 2008; Homewood et al. 2009). Women in Emangulai B therefore lack access to the people who have information on the land-allocation process. Many of them are still unaware of the steps involved in land allocation. The explanation below from a poor woman reflects this lack of knowledge about the land-acquisition process:

“I wait for the public message from the village council saying that land is going to be allocated. I join the others and go to Emangulai. I wait for my name to be called. I go one day and they do not call my name. I go the following day and still they do not call my name. I keep going right up to the last day. The process can go on for a week. And still they do not call my name. I have to wait until the next time a public message is sent and it can take a year. I have now gone through this process three times and still I do not have land.” (Esther, female, Emangulai B, individual interview)

According to a member of the Kirya village council, the first step would have been for Esther to travel to the village headquarters and fill out a prescribed form requesting land. Such information is passed on to people through informal social networks. Although Esther has been trying to get land for about 3 years, she has yet to complete this first step. Specific experiences vary, but the general lack of publicly available information on the land-allocation process leaves many poor men and women with no land on which to practice irrigation. Even when some women succeed in negotiating with the bureaucracy and obtaining a parcel of land, they tend to receive land that is far from water sources. Such land is euphemistically referred to as a ‘rain-fed’ plot, even though rainfall amounts are too low to support rain-fed farming in Kirya.

Not all land that the village council allocates for irrigation is considered prime irrigation land by the community. Community members consider land to be prime for irrigation purposes when it has a constant and reliable source of water. Pastoralist men and women who hold land rights to such desirable land are often challenged by non-pastoralists in the community who wish to have part of that land reallocated to them, both through lodging complaints with the village council and, when this fails, through the courts. Indeed, the VLA allows for need-based reallocation when one member of the community has a large piece of land while another has none. In this context, any man or woman with rights to land for irrigation must also have the knowledge required to protect the land from reallocation. Despite the broad requirement of the VLA that village councils treat men’s and women’s land claims equally, individual interviews reveal that men fare much better in maintaining rights to land than women do. Gender differences in the experience of maintaining land rights are reflected in the following two cases recounted to us during individual interviews.

Supeet is a wealthy male herder who is well connected to the village council and by extension, the general village administration social network. He serves on a village committee with some of the members of the village council. He currently lays claim to an atypically large piece of land (20 ha), much of which is ideal for irrigation. He uses some of the land for irrigation and some for dry season grazing. Recent migrants into Kirya have sought to have the land reallocated to them. According to Supeet, they first made the request for reallocation to the village council, and later went to the court system when the reallocation by the village council failed to happen. At the time of the fieldwork, Supeet had successfully defended his rights to this land, even after those challenging him appealed to authorities at the administratively more powerful district and regional levels. Supeet understands the land-acquisition process and draws on social networks to successfully navigate formal and informal legal institutions to maintain rights to his land.

Contrast Supeet’s experience with that of Nabulu, a widow from the same community who previously laid claim to 4 ha of land allocation, all of which was ideal for irrigation. Although Nabulu is not as wealthy as Supeet, she would still have been categorized as wealthy when she had access to 4 ha of prime land. Like Supeet, she frequently visits the village headquarters and also serves on a village committee with some village council members. Recent migrants also targeted her land for reallocation. Citing the VLA, the village council reallocated 2 of her 4 ha to the newcomers. She refused to vacate the land and continued to grow crops on it. Eventually, she was violently beaten, and her crops were burnt by the new owners. She made several attempts to reverse the village-level decision by appealing to the division- and district-level officials. None of her appeals bore fruit. One of the authors witnessed a polite confrontation between Nabulu and a senior member of the village council where she lamented the loss of her land and the failure of the village council to protect her claims to the land. The official responded by telling her that she lost the land because “it is the law and it must be followed.” Nabulu is trying to get back her claims to the land in the face of formidable obstacles that are positioned at he interface of access to knowledge and authority and which are further reinforced by social identity. She told one of the authors that “it is because I am a Maasai and a woman that they have taken my land.”

Engagement in irrigation farming has been identified by pastoralists and recent in-migrants as the most effective means of managing climate risk in this dryland community. The examples of Supeet and Nabulu demonstrate the multiple knowledge domains that people draw upon to pursue such preferred adaptive practices. Furthermore, the example demonstrates the intersection of gender and ethnicity in limiting access to knowledge about resource rights and access to the institutions that are mandated to ensure such rights. Successful adaptation requires not only the agronomic and technical knowledge that enables new economic activities (e.g., the practical knowledge of irrigation in a dryland environment), but also a social knowledge base that enables one to navigate new institutional arrangements. In Emangulai B, one needs to know the processes and institutions involved in land allocation, the formal and informal legal institutions for maintaining rights to land and, simultaneously, the informal social networks necessary to successfully navigate these processes. As such, access to knowledge is entwined with other mechanisms of access, particularly, institutions and authority, in shaping gendered vulnerability to climate change.

### Gendered access to fast-maturing seeds in Mangio

During the community workshop, switching to fast-maturing seed varieties was ranked second to education in its effectiveness in reducing climate risk. A full list of adaptive practices discussed at the workshop, their respective ranks, accessibility scores and effectiveness at the household can be found in Table S2. Although maize seeds are the most widely planted, some farmers also plant soybeans. People said that growing fast-maturing seeds enables them to reduce the risk of crop failure within the current constraint of a shorter rainy season. In the quote below, a participant summarizes the benefits of fast-maturing maize seeds over landraces:

“When we plant maize, specifically when we plant fast-maturing seeds, we harvest a lot of maize. And the maize is long and large. The cob is actually quite thin, it is the kernel that is large. The seeds we have used in the past don’t do well these days. When you plant, you get only a small harvest.” (Juma, male participant, Mangio village community workshop)

Fast-maturing seeds received an accessibility score of 4 at the community workshop, which means that most families in the village are able to access a certain amount of fast-maturing seeds. Men perceived the seeds to be more effective in averting climate risk than women did at the household level. As indicated in Table S2, the average score for men was 3.1 while that for women was 2.2; in households where both men and women said they made decisions together and were hence interviewed together, the average score was 2.8. These gendered differences are explained by qualitative data collected in the household and during individual interviews. Like irrigation in Kirya, fast-maturing seeds are promoted by national policies on agriculture and climate-change adaptation, but implemented by the local village administration (Muthoni and Wangui 2013). What emerges in Mangio Village is a highly differentiated access to seed technology. Similar to Kirya the poorest and women farmers are typically left out. Though all are Ugweno people, we have found that distribution of new agricultural technologies like fast-maturing seeds is highly concentrated among the wealthiest farmers most of who are male. The village administration charged with seed distribution openly favors wealthier farmers by giving them more seed than they give to poorer farmers. For example, key informants indicated that in 2011 wealthy farmers received 10 kg of maize seeds per household while poor farmers received only 2 kg. A key informant from the village administration justifies this by arguing that the wealthy are less likely to consume the seeds as food, and more likely to purchase additional inputs necessary for an optimal harvest such as fertilizer. Women farmers who managed to obtain seeds from the village administration explained how the officials treated provision of inputs as a favor rather than as a right.

In order to use fast-maturing seeds to reduce climate risk, farmers need not only to access seeds but also agronomic knowledge to take advantage of the properties of hybrid seeds. The top ranked adaptive practice during the community workshop was education (Table S2). Participants at the community workshop differentiated between two types of education, both of which are critical for effective adaptation. The first is formal education for children and youth so they can find high-paying off-farm employment outside the village. Second, and more relevant to this discussion is farmer education. During the workshop participants explained that without this education, farmers will not be able to perform any of the other adaptive practices discussed. The village administration is charged with organizing formal agricultural training workshops where agriculture extension agents teach farmers how to plant and care for the seeds in order to maximize yield. Access to the agricultural training workshops is skewed toward the wealthier male farmers since it is through their social networks and institutional connections that information about the workshops flows. As one man from a poor household indicated:

“My wife and I have not received any training from an extension officer. We know the extension officers in the area but they have not visited us for any training or advice. There are so many workshops for farming and cattle keeping but there are only certain people who are always invited. The village administration is in charge of inviting the people to attend those seminars. But we were not informed and we were left out.” (Maneno, male, Mangio village, household interview)

While knowledge does flow from some of these workshop attendants to other members of the community, such flows happen through existing social networks and still leave out most community members. Without this new knowledge, many, especially women call on their existing knowledge base to maximize productivity. Many use animal manure on the new seeds and call on traditional methods of predicting the start of the rains to plant the new seeds very early in the rainy season. Rather than an erosion of traditional knowledge reported in much of the TEK literature (Singh et al. 2010; McCarter and Gavin 2014), what we observe in Mangio are attempts to synthesize external knowledge where possible, while drawing upon and modifying established agricultural knowledge to better cope with changing social and climatic conditions.

Most farmers are excluded from the seed and knowledge acquisition channels set up by the village administration. They are predominantly women and the poor, and their alternative strategies are responses to this exclusion. One of the strategies they use involves seeking fast-maturing seeds from alternative sources. Those who can, buy the seeds available in local stores. A 2-kg bag of improved maize seeds costs about US$10 which is prohibitively expensive in Mangio. As is true in other farming communities, many farmers in Mangio rely on friends and family for the seed access (McGuire 2008). In Mangio, such seed networks are typically cultivated and maintained by women.

Farmers are also seeking access to land for irrigation in the lowlands about 1–2 h walk from the village. The demand for irrigated land overlaps with the use of fast-maturing seeds in two ways. First, people with fast-maturing seeds seek irrigated land so as to lower the risk of crop failure and the financial losses from the costly seeds. Second, those unable to obtain fast-maturing seeds also seek irrigable land to avoid crop failure associated with rain-fed farming. Seeking land for irrigation is a continuation of a process of downslope movement of farming that began with the breakdown of traditional common property use rights that restricted farming in low-lying wetlands within the village. These previously protected wetlands within the village were quickly absorbed by adjacent farms (Velempini et al. 2016). Currently farming is moving further down the altitudinal gradient where irrigation can be practiced. Access to these lands is maintained through arrangements made by farming women with predominantly male land owners. During household interviews, some poor women explained that they are able to receive free access to a small portion of irrigable land through existing relations with land owners. Others enter into a share-cropping arrangement where they pay a 90 kg bag of maize for 0.8 ha of land. Key informants said that when one uses appropriate fertilizer inputs, they are able to harvest 15 bags of maize on 0.8 ha of land in the irrigated lowlands. This fallback strategy relies on a highly gendered form of knowledge and resource access which is critical for coping with increased climate variability. Women use social networks and negotiation skills to obtain and maintain access to the lowland sites. Those unable to find fast-maturing seeds or land for irrigation face lower productivity given lower and increasingly sporadic rainfall (Velempini et al. 2016), as they either rely on landraces or plant seeds harvested from fast-maturing seeds which typically do poorly as indicated in the quote below:

“I planted fast growing maize three years ago and I have been replanting its harvest until it is no longer productive. When I planted it for the first time I had a good harvest but now I get very little.” (Rebeka, female, Mangio village, household interview)

The Mangio Village case study further illustrates that the contributions of local knowledge to adaptive practices are shaped by the dynamics of related mechanisms of access, notably access to technologies and institutions. Thus, women’s adaptive practices reflect both responses to changes in climate variability and to gendered forms of exclusion from land and technologies provided in the name of adaptation. While the distribution of external inputs such as fast-maturing maize seeds is by no means a panacea for managing increased climate variability, the potential for current and future components of planned adaptation to reinforce existing inequalities in access should be noted. Women in this study illustrate how people at the margins can work outside formal government structures that facilitate planned adaptation when such structures exclude them. Such work at the political and geographic margins of the village demonstrates the dynamic and heterogeneous characteristics of local knowledge as well as elements of continuity that draw upon existing elements of environmental, agricultural, and social knowledge in developing new adaptive practices.

## Conclusion

Rather than laying our focus narrowly on “traditional” forms of knowledge, we have located our focus on differentiated and dynamic local knowledge in the context of the contemporary set of adaptive practices identified as the most effective in reducing local climate risk. We assert that adaptive practices identified at the local level are the outcomes of gendered mechanisms of access that have created gendered dynamics of vulnerability and, consequently, gendered knowledge production to cope with climate and other stressors. In keeping with Rocheleau et al.’s (1996) notion of a “gendered science of survival,” we find that while established forms of culturally based knowledge inform the management of complex agro-ecologies, women’s strategies to build social networks, access resources, and gain access to formal institutions reflect equally important aspects of local knowledge. Whether in women’s downslope irrigated parcels in the North Pare highlands or district and village offices where women’s land claims are pursued and contested, “knowledge for adaptation” takes diverse forms. Indeed, dynamic forms of social knowledge required to gain and maintain access to land, technologies, and institutions are often a prerequisite for employing culturally transmitted, place-based forms of environmental knowledge necessary to manage increased climate variability.

The case studies raise the question of how adaptation policies and programs can engage with this broader knowledge domain by addressing the gendered mechanisms of access that limit adaptations options for many women. On the surface, the case studies suggest the need to ensure gender equity in the diffusion of technologies and other resources for adaptation. This would include adherence to equity principles enshrined in Tanzania’s land law. Preemptive efforts would also need to be made in order to decrease gender inequality of access to the suite of externally identified technologies and applied agricultural knowledge that are diffused in the name of climate-change adaption in rural Tanzania. Beyond this, adaptation to climate change will require consideration of options that extend well beyond existing adaptive practices and the standard menu of agricultural inputs. In order to avoid gender exclusive access and benefits, research that allows for gender-sensitive assessment of candidate technologies will have to expand considerably.

Knowledge production at the margins raises important questions about the prospects for knowledge integration. Such integration would fuse ostensibly distinct knowledge systems, local and external (alternatively traditional and scientific), in ways that enhance the larger knowledge base to guide planned adaptation. Community-based adaptation encompasses a suite of methods that seek to enable deliberative and inclusive approaches to adaptation and privilege local priorities for reducing climate risk by “unprivileging” external scientific knowledge. Within CBA, efforts to integrate local and scientific knowledge should recognize the limits of an extractive approach that would isolate techniques of local agro-ecological management from the broader knowledge system for incorporation into adaptation projects. By avoiding a truncated notion of local knowledge, such efforts can learn from the changing social knowledge base that has developed to cope not only with greater climate variability but equally the limited resource options that stem from unequal access.

In short, while there is much to be learned from the ingenuity and innovative practices that have enabled women to cope with greater climate variability, our understanding of them should never be divorced from the gendered forms of exclusion that shape knowledge–practice complexes. Despite the dangers of simplifying or essentializing local knowledge systems, CBA may provide platforms for envisioning of alternative futures in ways that are transparent, inclusive, and make possible anticipatory forms of learning that are sensitive to gender differences. In our view, engagement with gendered local knowledge and the gendered mechanisms of access that shape them is not only possible, but a critical task if we are to rework technocratic and incremental visions of climate-change adaptation in ways that realize its transformative potential.

## Electronic supplementary material

Below is the link to the electronic supplementary material.

Supplementary material 1 (PDF 55 kb)
