# Supplementary material for: Gendered knowledge and adaptive practices: Differentiation and change in Mwanga District, Tanzania
Source: Ambio. 2016 Nov 22;45(Suppl 3):276–86. doi: 10.1007/s13280-016-0828-z (PMC5120021; doi:10.1007/s13280-016-0828-z)
Supplement: Supplementary file 1 — Supplementary material 1 (PDF 55 kb) [file 13280_2016_828_MOESM1_ESM.pdf]

Ambio

Electronic Supplementary Material

This supplementary material has not been peer reviewed

Title: **Gendered Local Knowledge and Adaptive Practices: Differentiation and Dynamism in Mwanga District, Tanzania.**

Authors: Thomas A. Smucker and Elizabeth Edna Wangui

The tables below show the perceived effectiveness in reducing climate risk in two villages in Tanzania. Rank 1 is the adaptive practice that is perceived to be most effective. Accessibility was assessed on a five point Likert scale where 1 meant only a few people practiced the activity.

Table S1 Effectiveness of climate change adaptation practices in Emangulai B sub-village

Adaptive Practice	Effectiveness at reducing climate risk at community level		Effectiveness at reducing climate risk in the household	
	Rank	Accessibility	Male	Female
Practice irrigated farming	1	3	1.3	1.6
Rely on a combination of herding and farming	1	3	1.6	1.9
Combine traditional and improved breeds	2	1	2.3	1.7
Educate children	3	5	2.8	3.1
Treat livestock with modern veterinary medicines	4	5	4.1	3.5
Engage in livestock and other	5	3	0.8	0.2

trade				
Engage in temporary employment	5	3	0.4	1.1
Move livestock	6	5	3.4	2.5
Buy fodder	6	5	2.7	3.8

Table S2 Effectiveness of climate change adaptation practices in Mangio village

Adaptive Practice	Effectiveness at reducing climate risk at community level		Effectiveness at reducing climate risk in the household		
	Rank	Accessibility	Male	Female	Both
Education	1	4	4.4	4.0	3.2
Using fast growing seed varieties	2	4	3.1	2.2	2.8
Early planting	2	4	3.1	3.1	2.6
Applying animal manure	2	4	4.6	2.8	4.1
Farming root crops	3	2	3.4	3.5	3.6
Using improved livestock breeds	4	4	3.9	3.5	3.5
Less forest fires	5	4	4.5	5.0	4.7
Planting trees	5	4	2.6	3.7	4.2
Terracing	5	4	0.6	0.0	0.7
Participating in village community banks	6	2	2.4	2.5	2.3
